# Huntington's Disease: An Immune Perspective

**DOI:** 10.1155/2011/563784

**Published:** 2011-08-24

**Authors:** Annapurna Nayak, Rafia Ansar, Sunil K. Verma, Domenico Marco Bonifati, Uday Kishore

**Affiliations:** ^1^Centre for Infection, Immunity and Disease Mechanisms, Biosciences School of Health Sciences and Social Care, Brunel University, West London UB8 3PH, UK; ^2^Centre of Biotechnology and Bioinformatics, School of Life Sciences, Jawaharlal Nehru Institute for Advanced Study, Secunderabad, Andhra Pradesh, India; ^3^Centre for Cellular and Molecular Biology, Uppal Road, Hyderabad 500 007, India; ^4^Unit of Neurology, Department of Neurological Disorders, Santa Chiara Hospital, Largo Medaglie d'oro 1, 38100 Trento, Italy

## Abstract

Huntington's disease (HD) is a progressive neurodegenerative disorder that is caused by abnormal expansion of CAG trinucleotide repeats. Neuroinflammation is a typical feature of most neurodegenerative diseases that leads to an array of pathological changes within the affected areas in the brain. The neurodegeneration in HD is also caused by aberrant immune response in the presence of aggregated mutant huntingtin protein. The effects of immune activation in HD nervous system are a relatively unexplored area of research. This paper summarises immunological features associated with development and progression of HD.

## 1. Introduction

Huntington's disease (HD), first discovered by George Huntington in 1872, is an autosomal-dominant inherited progressive neurodegenerative disorder that affects control over movement and cognition along with the development of psychological symptoms [1]. The prevalence of the clinical syndrome is 3–7 : 100000 whereas nearly 20 : 100000 are carriers of the gene responsible for the disease. Symptoms include weakening of mental abilities leading to a change in personality (i.e., depression, suicidal tendencies, and in rare cases, violent behaviour), development of dementia, loss of psychomotor functions due to lack of muscle coordination, and abnormal sudden jerky involuntary movements collectively called chorea that heavily affect gait and agility [2]. Although the disease has the potential to present itself at any time from childhood to old age, it is characterised by the onset of midlife chorea (around 33–44 years of age) [2]. 

HD is caused by an abnormal expansion of otherwise normal CAG trinucleotide polyglutamine repeats (polyQ repeats) on the N terminus of the *IT 15* (*Htt*) gene, as it codes for the protein huntingtin (Htt), which was discovered in 1993 and is located on chromosome 4p16.3 [3]. Htt is abundantly expressed in the brain and testes with moderate expression observed in other organs such as liver, heart, and lungs [2]. Even though the complete function of Htt still remains unclear, it has been observed to be involved in cytoskeletal anchoring and transport of mitochondria along with vesicle trafficking to mediate endocytosis, thus implicating in embryogenesis and development [4, 5]. The deficiency of this protein leads to embryonic lethality in HD knockout mice [6]. Interestingly, Htt has also been shown to be of importance during the postembryonic stage including craniofacial development, forebrain formation especially the cortical and striatal areas and also in survival of neurons [5, 7]. The active participation of Htt in brain development and maintenance therefore illustrates the importance of Htt in the CNS.

In an HD patient, the abnormally amplified CAG repeats in the Htt gene lead to transcription of the mutant huntingtin (mHtt). The intensity of the disease progression is directly related to the number of these CAG repeats. For example, in a normal person, the number of CAG repeats is approximately 8–39, whereas, in HD, the repeats can range between 36 and 120 in number [8]. This aberrant expansion of polyglutamine repeats is also implicated in development of neuronal dysfunction which is consistent with the wild type Htt function. This dysfunction contributes to the manifestation of clinical symptoms. Although the mechanism through which mHtt gains toxicity through gain-of-function still remains under debate, the CAG repeat results in aggregation of inclusion bodies containing fibrillar mutant Htt fragments within the striatal neurons. These abnormal aggregates appear to be the main cause of neurodegeneration in the disease [9]. During the expression of mHtt, the first 100–150 residues including the polyglutamine repeats are cleaved off and these fragments are toxic [10]. 

The neuropathological hallmark of HD is the degeneration of the nuclei of the basal ganglia situated in the lateral ventricle brain, that is, the caudate nuclei. The intensity of the degeneration can vary from mild to severe in the caudate nuclei while it is less prominent in the putamen. As the disease progresses, there is a dramatic increase in neuronal loss from caudate along with presence of both reactive astrocytes and microglia in the grey matter in the caudate as opposed to the early stages in which no significant gliosis is observed [11, 12]. In the neostriatum, mutant Htt is found in the cell bodies and synaptic processes of surviving neurons and glial cells. Interestingly, the expression of Htt is not limited to just the brain but is an ubiquitously expressed protein [13].

## 2. Immune System in Brain

The brain was once thought to be an immunologically privileged organ with an inability to generate humoral and cellular immune responses. The presence of a blood-brain barrier (BBB) further supported this idea as it separates the CNS from the rest of the systemic immune system. However, this case holds true for a healthy CNS that has not been affected by any immune or inflammatory disorder. In the event of trauma, axotomy, ischemia, and degeneration, the immune system within the CNS gets activated. Microglial cells are resident macrophages of the CNS that are quiescent in a healthy brain. The activation of microglial cells leads to upregulation of their cell surface antigens and secretion of proinflammatory cytokines [14]. This secretion of cytokines such as IL-6, IL-12, and TNF-*α* leads to a dialogue between the neighbouring microglial cells, astrocytes, T-cells, neurons, and myeloid progenitor cells. Along with chronic inflammation, the secretion of these proinflammatory cytokines can lead to free radical production [15], NMDA-mediated excitotoxicity [16], and caspase activation [17], causing widespread damage in the brain due to neuronal death.

## 3. Immune System in the Huntington's Disease Brain: Complement and Cytokines

The presence of a pathological injury within the brain can initiate an immune response. In most neurodegenerative diseases, an immune response to the abnormal folding of proteins and aggregates triggers neuroinflammation which in turn is implicated in neuronal degeneration. Consistent with the origin of neuroinflammation and neurodegeneration in other neurodegenerative diseases [18], mutant Htt aggregates are observed and neostriatal atrophy is a striking feature in HD brains which suggests massive neurodegeneration in the neostratum, that is, the putamen and caudate [19]. The pathogenic protein aggregates are foreign bodies to the otherwise immunoprivileged organ and the antigen presenting cells of the local immune system, that is, microglial cells are likely to recognise the aggregates. It is also possible that protein aggregates cause neuronal death through apoptosis, and apoptotic bodies can activate microglia and the CNS innate immune system. The progression of HD still remains poorly understood. However, premanifest HD, where the carriers of the gene do not exhibit the classical signs and symptoms of the typical HD patient, provides important clues to the disease progression. These include the presence of activated microglia in the striatum as a result of mHtt aggregation and early neuronal dysfunction including elevated pathogenic extrasynaptic NMDA receptor signaling, reduced synaptic connectivity, and loss of brain-derived neurotrophic factor (BDNF) [20]. There is also an increase in the expression of inflammatory cytokines see below.

As mentioned earlier, the resident macrophages of the brain, microglial cells supervise their microenvironment for any sign of trauma, injury, or foreign bodies. In the presence of these stimuli, activation of microglial cells takes place. Presence of activated microglial cells is a good marker for immune activation. A recent study reported microglial activation in presymptomatic HD gene carriers and also an association between the activation and striatal neuronal dysfunction [12]. This study is supported by an earlier study in which activated microglia was found throughout the affected areas in HD and the intensity with which it accumulated coincided with the grade of disease progression [21]. This accumulation of microglia leads to a series of events before it ultimately leads to neuronal loss. However in HD, the precise mechanism by which the mutant Htt could lead to region-specific neuronal death is still unclear. A number of studies have suggested a role of immune components that might initiate gliosis and neurodegeneration [22–24].

**Figure 1:**
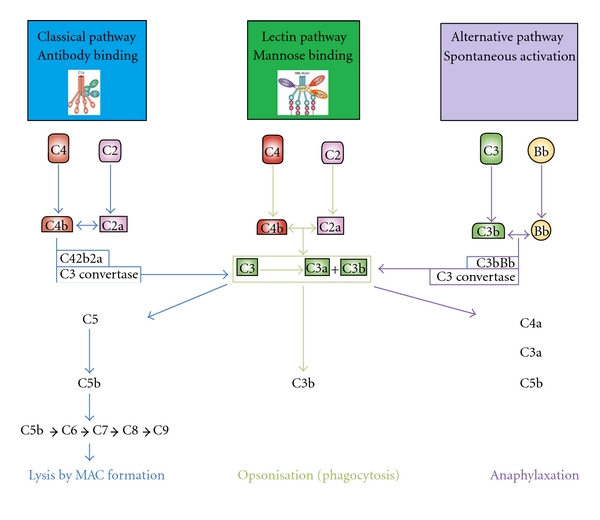
The complement system: the classical complement pathway involves a sequentially acting multistep cascade in which the complement components C1q, C1s, C1r, C4, C2, and C3 play important roles. C1r and C1s, the two serine protease proenzymes, along with C1q constitute C1, the first component of the classical pathway [25]. The activation of the C1 complex (C1q + C1s–C1r–C1r–C1s) subsequently activates the complement through the cleavage of C4 and C2 to yield the central molecule C3 convertase that cleaves C3, leading to the activation of the C2–C9 components and thus the formation of the terminal membrane attack complex (MAC) [26]. The MAC binds to cell membranes and facilitates cell lysis. The alternative pathway is initiated by low-level activation of C3 via its hydrolysis (C3b) and activated factor B. The activated C3b binds factor B that is cleaved by factor D to form C3 convertase. The main difference between classical and alternative pathway is that the initiation of alternative pathways is not dependent on the presence of immune complexes. The lectin pathway is activated following the recognition and binding of pathogen-associated molecular patterns (PAMPs) by mannose-binding lectin (MBL) [27]. The binding of MBL to repetitive carbohydrate patterns on pathogen surfaces has the potential to activate the lectin pathway through the MBL-associated serine protease (MASP), designated as MASP-2, that in turn leads to the activation of complement components C4, C2, and C3. The association of MBL-MASP complex of the lectin pathway is analogous to C1 complex of the classical complement pathway.

One of the main immune mechanisms involved in the immune surveillance of the CNS is the complement system that is activated upon stimulation by pathological peptides such as mutant Htt. The complement system is a key factor in several neurodegenerative diseases and is the most important and powerful humoral component of the innate immune system [28]. The vital functions of the complement system include host defence against the action of pathogenic microorganisms, removal of immune complexes and apoptotic cells, and facilitate adaptive immune responses [29]. It also mediates the production of anaphylatoxins (C3a, C4a, and C5a) that trigger degranulation, cell lysis, and phagocytosis via induction of chemotaxis and cell activation [29]. Complement system gets activated via three pathways depending on target ligands and the recognition complement component. However, the common aim of all the three pathways is to activate the central component of the complement system, that is, C3 (Figure 1). Altered levels of the activation of the complement system are considered important causative factors in inflammatory, neurodegenerative, and cerebrovascular diseases [28, 30, 31].

Expression of a number of complement components such as C1q, C1r, C4, C3, as well as the complement regulators, C1 inhibitor, clusterin, MCP, DAF, CD59 in HD brain samples with severe atrophy, has suggested the recruitment of the complement system in the HD pathogenesis [23]. 

Research into the involvement of the peripheral nervous system in HD is still in its early days. Recently the upregulation of proinflammatory cytokines such as IL-6, IL-8, and TNF-*α* was detected in the peripheral nervous system HD patients, irrespective of the disease stage [22, 32]. Proteomic profiling of plasma from HD patients also detected upregulation of proinflammatory cytokines (especially IL-6) along with other innate immune proteins such as the acute-phase protein *α*_2_-macroglobulin (*α*_2_M) and clusterin [32]. Clusterin is a multifunctional glycoprotein that is involved in diverse mechanisms of cytoprotection, membrane recycling, and regulation of membrane attack complex. Upregulation of clusterin is also implicated in a variety of physiological and pathological states including apoptosis and response to injury [32]. Clusterin has been previously associated with other neurodegenerative disorders such as Alzheimer's disease (AD) where its plasma level correlates with the degree of neurodegeneration [33]. Interestingly, clusterin has also been found to be expressed in both the peripheral plasma and in CSF thus suggesting widespread immune activation [32]. Up-regulation of *α*_2_M is seen in the peripheral plasma and its release is stimulated by pro-inflammatory IL-6. These findings are consistent with observations in AD in which *α*_2_M is upregulated in reactive astrocytes and is also observed to bind to A*β* which is the pathogenic peptide responsible for the formation of senile plaques in AD [34]. Thus, a simultaneous upregulation of immune proteins and cytokines is evident in both the central and peripheral nervous system in HD.

## 4. Neuroinflammation and Neurodegeneration in HD

**Figure 2:**
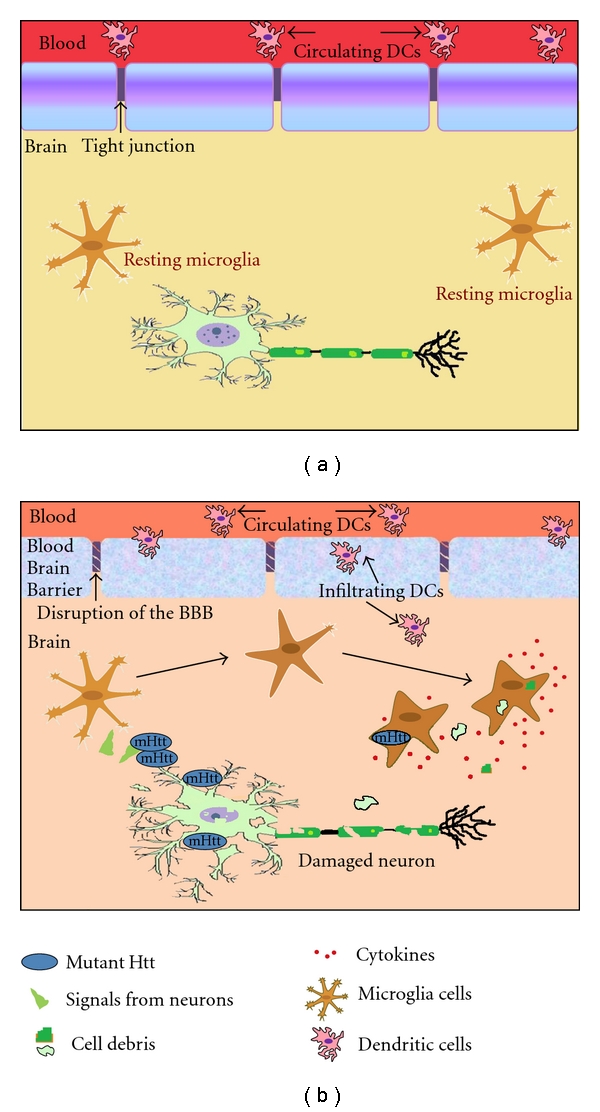
Comparison of immune activation in normal brain (a) versus HD brain (b). The normal brain is in a state wherein the microglia cells are in a resting state and are surveying its microenvironment. The BBB protects the brain from circulating immune cells such as DCs as well as pathogens and other foreign material. However, in the HD brain, the damaged neuron has aggregated mHtt and this activates the resting microglia cells and hence leads to phagocytosis of the cell debris along with secretion of proinflammatory cytokines that leads to neuroinflammation. This neuroinflammation weakens the BBB leading to infiltration of DCs and further elicits the immune response. Microglia cells that express mHtt also contribute to the inflammation and degeneration of the brain cells.

Glial cells constitute nearly 90% of the total brain cells, and their main function is to provide neurons with nutrition, growth factors, and structural support. These cells are also responsible for maintaining the normal physiology within the CNS. The normal brain is devoid of any immune activation on a day-to-day basis. However, a series of events and cascades due to infections, trauma, toxins, and stroke can lead to the pathological neuroinflammation and neurodegeneration that are seen in most neurodegenerative diseases, including HD [18, 35]. Neuroinflammation is seen as a double-edged sword, that is, having both beneficiary and harmful effects. Acute neuroinflammation is helpful in elimination of toxins and necrotic cells, and it is the more beneficiary type of inflammation within the CNS. Microglia are stimulated by these substances and takes on a phagocytic role along with the secretion of various cytokines and chemokines. However, this type of inflammation is a short-lived phenomenon. Despite the oxidative and nitrosative stress, the process is seldom harmful to long-term neuronal survival. Hence, it minimises further damage to the brain cells along with repairing damaged tissue. On the other hand, chronic neuroinflammation is associated with exacerbating neuronal damage. Chronic neuroinflammation includes not only the extended activation and proliferation of microglia but also increased secretion of proinflammatory cytokines and increased superoxide and nitric oxide production. This prolonged inflammation affects the BBB which in turn supports the infiltration of macrophages and myeloid progenitor cells into the brain parenchyma that further intensifies the already augmented inflammation. Thus, the detrimental effects of chronic inflammation are highlighted in neurodegenerative diseases as opposed to the beneficial acute inflammation (Figure 2). 

Under normal conditions, microglial cells are in a quiescent state with extremely sensitive spidery processes monitoring the microenvironment around them and lack MHC class I and II proteins. Upon activation, these cells take up the role of antigen presenting cells and in turn activate T cells. Activated microglia play an important role in the development and progression of HD as well as other neurodegenerative diseases like AD and Parkinson's disease [18, 36]. Several studies have examined the association of microglial activation with progression of HD. A postmortem study of HD brains revealed significant accumulation between activated microglia in regions affected by HD, especially the basal ganglia and the frontal cortex [21]. Interestingly, the density of activated microglia correlated and the severity of HD pathology, suggesting a pronounced association between microglial activation and subsequent neuronal death [21]. Tai et al. were the first to report evidence of activated microglia in the brains of presymptomatic HD individuals and striatal neuronal dysfunction [12]. The presence of reactive microglia was also seen in early manifest HD, the intensity of which again consistently increased with the progression of the disease [21]. Positron emission topography (PET) imaging studies on presymptomatic, manifest, and post mortem brains have positively reported different intensities of microglial activation that correlates with disease progression; however microglial activation in a minority of presymptomatic HD brains was undetectable due to the possibility of the patients being further away from developing the disease [11, 37]. 

Whether inflammation in the CNS is in response to neuronal death caused by the toxic mutant Htt or vice versa is debatable. In the affected areas of the brain, the expanded mutant Htt is found in the form of N-terminal fragments, oligomers, and polymers (instead of intact monomeric form), mostly accumulating in the cortex [38]. Mutant Htt is known to be a key factor in promoting inflammation in HD, either directly or indirectly. However, the question now arises if the inflammation is brought about by mutant Htt expressed in the milieu or by inflammation due to the activated microglia. The increased expression of the immune molecules along with the secretion of proinflammatory cytokines is an abnormal state for an otherwise immunoprivileged brain, and the widespread inflammation in HD is evident through the upregulation of cytokines both in the CNS and the peripheral plasma. 

Studies to assess the toxicity of mutant Htt and its association with neurodegeneration and neuroinflammation have been carried out in recent years. Expression of mutant Htt in glial cells, including astrocytes and microglia, has been implicated in supporting the inflammation observed in HD. Mouse models of HD (R6/2 transgenic mice) that exhibit a HD phenotype have shown expression of mutant Htt within glial cells, especially in astrocytes. The intranuclear expression of mutant Htt reduced the neuroprotective function of astrocytes, rendering neurons vulnerable to degeneration [39, 40]. The glial cells are also known to protect neurons against excitotoxicity by clearing excess excitatory neurotransmitters from the extracellular space [41]. This intranuclear expression of mutant Htt has been suggested to lead to degeneration of the medium sized spiny neurons in the striatum of the HD brain [39] and also exacerbate the neurological symptoms [40]. In addition, mutant huntingtin aggregates within neuronal cells have been identified using immunofluorescence techniques [38]. Interestingly, the N-terminal mutant Htt in glial cells promoted the death of neurons (neurons that did not express mutant Htt) [39]. 

Neuroinflammation is also caused by the release of cytokines and chemokines from the microglial cells as described earlier and the subsequent respiratory burst and oxidative stress. The overall inflammatory response seen in HD may occur simultaneously in the CNS and in the periphery or begin peripherally and spread centrally across the BBB or vice versa, involving immunomodulatory molecules [22]. One such association is of C5a, which activates phagocytes and local mast cells to release their granules containing histamine and TNF-*α*. The cytokines secreted by macrophages include IL-6, IL-1*β*, IL-12, and TNF-*α*, and certainly the upregulation of IL-6 in HD patients has been identified in the periphery [22]. TNF-*α* stimulates the migration of dendritic cells carrying the mutant Htt protein aggregates from peripheral tissues to lymph nodes and maturation which would initiate the adaptive immune response which in turn augment a peripheral immune response [22]. This infiltration of the immune cells from the peripheral plasma into the CNS might be due to the weakening of the BBB preceded by neuroinflammation. A study using RT-PCR and in situ hybridisation revealed that the classical pathway components including C1q (C chain), C1r, C4, C3, as well as the complement regulators are expressed at much higher level in HD brains compared to normal brain. Complement activation on neurons and increased biosynthesis of C1q, the initiation subcomponent of the classical complement pathway, by microglia [23] suggests the possibility of the activation of the classical complement pathway contributing to the neuroinflammation. Whether this complement activation is due to the mutant Htt aggregation or due to apoptotic/necrotic neuronal cells is still unclear. In general, the widespread neuroinflammation contributes to neuronal death and vice versa, leading to progressive memory and motor function loss.

## 5. Perspectives and Conclusion

HD is a progressive age-dependent neuropathological disorder in which the immune activation is seen to be predominant in both the CNS and the periphery. The mutant Htt aggregates appear to trigger neurodegeneration and chronic neuroinflammation, which correlates well with HD clinical syndromes. Studies that would identify novel immunological biomarkers and their levels within the plasma and CNS can help monitor the progression of the disease. A potential upregulation of complement in premanifest HD is likely to be a useful immune biomarker in monitoring disease progression.

An important role of the process of autophagy has been shown to be involved in HD. Autophagy is a cell homeostatic mechanism of intracellular degradation to ensure continuous turnover of the intracellular components. This involves formation of autophagosomes, the double membrane structures, which fuse with the primary lysosomes. The contents of these autophagosomes are degraded in the lysosomes and either disposed off or recycled back to the cell [42]. In addition to housekeeping cellular function, autophagy is also considered important for the degradation of aggregated proteins. In HD, Htt-containing vacuoles display the ultrastructural features of early and late autophagosomes. In vitro, Htt-containing cytoplasmic vacuoles contain the lysosomal enzyme cathepsin D, thus suggesting the role of autophagy in HD [43]. The removal of both soluble and aggregate forms of mutant Htt via autophagy appears to be a neuroprotective mechanism [44]. However, a recent report of a slower and inefficient degradation of mutant Htt due to possible defect in autophagy may be one of the mechanisms of mutant Htt accumulation and subsequent neuroinflammation in HD [45, 46]. The contribution of the process of autophagy in the pathogenesis of HD needs further research. Studies involving the control of neurodegeneration and neuroinflammation in the CNS as well as the periphery by decreasing the upregulation of the inflammatory immune proteins as well as cytokines would pave way for a better prognosis of patients affected by HD. This could be made possible by developing therapeutic agents that target the complement and microglial activation, which in turn, would inhibit the concomitant release of proinflammatory cytokines.

## Abbreviations

HD: Huntington's disease
APC: antigen presenting cells
A*β*: amyloid *β* (1–42) peptide
AD: Alzheimer's disease
TNF: tumour necrosis factor
DC: dendritic cells
immDC: immature dendritic cells
MHC: major histocompatibility complex
CNS: central nervous system
Htt: Huntingtin
MSN: medium spiny neurons
MCP: monocyte chemotactic protein
DAF: decay accelerating factor
CSF: Cerebrospinal fluid.
